# The Definition of Puerperal Fever

**Published:** 1895-03-16

**Authors:** 


### The Definition of Puerperal Fever.

It has been said by many that the inclusion of puerperal fever in the list of notifiable infectious diseases is of no use in consequence of the want of any authoritative definition of the term; and in truth there is much reason to believe that as regards this particular disease the public does not get that measure of protection from the Act which by a slight modification of its terms it might be made capable of giving. The present position of affairs is a curious instance of difficulties arising from increasing knowledge. "Puerperal fever" is a crude and unscientific term, the meaning of which it is difficult to define unless we are content with saying that it is a fever occurring after childbirth, ending frequently in death. But modern science has taught many things, and among them that the wide group of deaths from "fever" after confinement contains deaths from many very different causes. There may be a local septic inflammation; there may be secondary local inflammations or abscesses, from either of which there may be septic absorption causing septicæmia or sapræmia; there may be phlebitis or even true pyæmia; there may be that condition which is spoken of as "peritonitis," or there may be that rapidly fatal form of toxic fever to which alone some men would apply the term puerperal fever. Then the modes by which these conditions actually kill, or, at any rate, the coarse pathological changes which take place, may take the form of a pneumonia, or a peritonitis, or other more or less localised inflammation. It thus happens that in the registration of the cause of death there is much opportunity for such cases to be lost sight of, unless it is continually borne in mind that under whatever name the disease is entered there is a strong presumption that any case of death within a fortnight of confinement is due to septic mischief of some sort. As regards the Notification Act, however, things are different. No medical man is bound to notify until he becomes aware that his patient is suffering from one of the dangerous infectious diseases mentioned in the schedule. Now about the ordinary fevers there is no great difficulty, except when sanitary authorities endeavour to enforce notification at a too early stage of the disease, before satisfactory notification is possible. But puerperal fever is another matter. On the one hand, there is great uncertainty in many men's minds as to the correct definition of the term, if, indeed, a definition be possible; on the other hand, certain teachers, by laying down a rule that medical men after having such a case should abstain for a lengthened period from obstetric work, have practically penalised the admission of having attended a case of "puerperal fever." Under such circumstances it is not to be wondered at that the notification of this disease has had such small effect. Two reforms are necessary. First, a definition of the disease which shall include all cases of pyrexia after childbirth, from whatever cause; second, an authoritative statement as to the exact process of disinfection which shall allow a practitioner to return again to his work. It is a complete anachronism, in view of what we know of antiseptic work in general surgery, that it should still be considered that time is a purifying agent, or that abstention from practice for long periods after attending a case of puerperal fever should be looked on as either obligatory or useful.

